# *APOE* Alleles Modify Symptom Onset in Early‐Onset Alzheimer's Caused by the *PSEN1* A431E Mutation

**DOI:** 10.1002/alz70855_097839

**Published:** 2025-12-23

**Authors:** César A Valdez‐Gaxiola, Frida Rosales Leycegui, Martha Patricia Gallegos Arreola, Sofia Dumois‐Petersen, Luis E Figuera

**Affiliations:** ^1^ Universidad de Guadalajara, Guadalajara, JA, Mexico; ^2^ Instituto Mexicano del Seguro Social, Guadalajara, JA, Mexico

## Abstract

**Background:**

Alzheimer's disease (AD) is the most common neurodegenerative disorder, characterized by neuropathological hallmarks such as amyloid β‐protein plaques, leading to neuronal loss. Early‐Onset Alzheimer's Disease (EOAD), which manifests before the age of 65, is a rare condition often associated with specific genetic variants. Patients carrying the *PSEN1* A431E mutation typically present with symptoms at an average age of 42.5 years, with rapid disease progression leading to death within an average of 7.5 years. This mutation represents the second‐largest population of EOAD cases caused by a single mutation. Given the clinical variability observed among affected families with the same mutation, we investigated the influence of additional genetic factors, such as *APOE* alleles, on disease phenotype.

**Method:**

A total of 89 patients with familial EOAD were evaluated at the CMNO‐IMSS genetics service. Autosomal dominant inheritance patterns and clinical features consistent with AD were identified. The *PSEN1* A431E mutation was confirmed by Sanger sequencing, and *APOEε2* and *APOEε4* alleles were genotyped using real‐time PCR. Data analysis included descriptive statistics, Kruskal‐Wallis, and simple linear regression to assess clinical correlations.

**Result:**

A significant association was identified between the age at symptom onset and *APOE* alleles. *APOEε4* carriers showed a delayed symptom onset of 4.15 years (*p* = 0.00035), whereas *APOEε2* carriers showed an earlier onset of 2.34 years (*p* = 0.037). However, no association was observed between *APOE* alleles and the nature of the first clinical manifestation.

**Conclusion:**

This study highlights an apparently inverse effect of *APOEε4* and *APOEε2* in EOAD patients with the *PSEN1* A431E mutation, compared to late‐onset AD. Specifically, *APOEε4* delays the age of symptom onset, whereas *APOEε2* accelerates it. These findings suggest that *APOE* alleles play a dual role as genetic modifiers in EOAD, offering potential avenues for personalized risk assessment and targeted interventions.

